# Supplementary material for: Lay community mental health workers (cadres) in Indonesian health services: A qualitative exploration of the views of people with mental health problems and their families
Source: PLoS One. 2023 Nov 9;18(11):e0289943. doi: 10.1371/journal.pone.0289943 (PMC10635455; doi:10.1371/journal.pone.0289943)
Supplement: Attachment — Submitted filename: Response to Reviewers.docx [file pone.0289943.s001.docx]

May 21, 2023

Dear Editor-in-Chief of PLOS ONE

Thank you for your email dated March 24, 2023, and reviewer comments for the manuscript titled **“Lay community mental health workers (cadres) in Indonesian health services: A qualitative exploration of the views of people with mental health problems and their families” (Manuscript ID: PONE-D-22-34567)**. As directed by your correspondence, we are submitting a substantially revised manuscript with three columns of tables that respond to each point raised by the academic editor and reviewer. We also submit a marked-up copy of our manuscript highlighting changes made to the original (track changes version) and an unmarked version of our revised paper without tracked changes. So again, we thank you for the opportunity to submit this revised manuscript. We look forward to your feedback.

**Response to the reviewer comments**

| **Comments** | **Response** | **Row** |
| --- | --- | --- |
| **Reviewer 1** |  |  |
| The current qualitative study conducted on a small sample aimed to explore the experiences of people with mental disorders and their families about the role of mental health workers in improving users’ mental health and in providing support to their caregivers. Given the relevance of roles assumed by families in the context of community psychiatric care in looking after relatives affected by mental disorders, the paper provides scientific contribution to the panorama of studies investigating on the role of family members in caring for relatives affected by mental disorders.  Globally, the clarity of the manuscript is overall good enough in the language style, syntax, and sentences construction. The topic is interesting enough and qualitatively good to be published. However, some changes and explanations to improve quality of manuscript for his acceptance should be done. | We thank the reviewers for taking the time to assess our manuscript.  We are grateful for the appreciation given by the reviewers for our manuscript.  We have ensured to improve the manuscript based on suggestions and input from reviewers. | - |
| **Abstract section** | | |
| In the description of the methods, the authors should also report the site or sites of recruitment and the mental disorders that predominantly characterized the sample. | We have added predominantly characterized mental disorders and sites of recruitment in the abstract.  In this study, the mental health problems experienced by participants were schizophrenia (100%/19 participants). Nineteen people with mental health difficulties are people diagnosed with schizophrenia.  The recruitment sites in this study are three provinces in Indonesia: West Java, East Java, and Aceh. | **Abstract**  Row 39 - 43 |
| In the results section, the authors could report the most significant data, in quantitative terms (relevant statistical data). | We have added the most significant theme in the quantitative term.  "The most significant theme was emotional support provided by cadres in terms of reception from cadres about people with mental health problems (59.1%), tangible support in which cadres help people with mental disorders get treatment (52.27%), and cadre roles as duties/mandates (51.36%) was factors that facilitated the success." | **Abstract**  Row 47 - 51 |
| The keyword "caregiver or “caregivers " could be entered. | We have inserted the new keyword "caregiver." | **Abstract**  Row 64 |
| **Introduction section** | | |
| In the ‘introduction section’ the bibliography is appropriate and updated.  However, it would be useful to mention, for example, some studies that have focused on the role of 1) the family as a resource for the rehabilitation process of the family member suffering from mental disorder, and of 2) the scientific evidence based interventions.  There are several evidences of the effectiveness of psychoeducational interventions involvement of family members aimed at mitigating the effects of the disease, in particular, schizophrenia (1,2,3,4,5,6 listed below).  The authors should integrate the bibliography reported, considering the development of EBM psychoeducational interventions aimed at family members of people with serious and disabling mental illnesses.  For example, a very recent study conducted on 136 caregivers showed that caregivers’ personal growth was associated with good family functioning, but also, adequate professional support. suggesting as the many challenges and positive aspects associated with caregiving they should be recognized by mental health services and integrated into routines clinical assessment and intervention framework. | We have added a paragraph explaining the reviewer's suggestions regarding the family as a resource for the rehabilitation process of people with mental disorders, evidence of the effectiveness of psychoeducational interventions aimed at family members, and suggested challenges and positive aspects associated with caregiving by integrated mental health services. | **Introduction**  Row 78-91 |
|  |  |  |
| Row 74. In the sentence “…….arrangements with families and the wider community in a therapeutic capacity..” it is unclear what the authors mean by ‘arrangements’. | What we mean is a collaboration with families and communities.  We have rearranged the sentence to  "Integrative collaboration with families and the wider community can improve mental health services in a therapeutic capacity." | **Introduction**  Row 78 - 79 |
| **Materials and methods section** | | |
| Row 135: The authors contextualize this study in a "larger research project"; the authors could clarify better and report more information on the mentioned project. | This study was part of a larger research project titled Exploration of the Role of Mental Health Workers (Cadres) in Community Mental Health Services in Indonesia, which explored the experiences of families and people with mental disorders who received support from mental health cadres.  We have inserted the title of the project in the manuscript. | Materials and methods  Row 158 - 159 |
| Row 148: The authors specify the countries in which the recruitments were carried out, but neither the structure in which the patients were recruited nor the number of accesses and the percentage of patients recruited on the basis of them is specified. | The participant selection process was carried out based on the following process:  We asked permission from the local government for each site (West Java, East Java, and Aceh) and coordinated with the Provincial and Regional Health Office. The health office directed us to the community health center for the research location. The Head of the community health center identified participants according to predetermined inclusion and exclusion criteria. Then the Head of the community health center, helped by a mental health cadre, made an appointment for a meeting between the participants and the researcher. | Materials and methods  Row 180 - 187 |
| Rows 149-150. The authors report “…… with details for 149 family participants and people with mental disorders presented in table 1.” Socio-demographic and clinical data of the sample is not present in the manuscript. Furthermore, Table 1 reports “Distribution of Focus Group Discussion Family and Client”. Could the authors clarify this inconsistency? It would be appropriate to report the clinical data of the sample (patients and family members), the diagnoses, as well as the role of the caregivers (i.e. mother, father, husband, wife, brother, sister, etc.) either in the table or in the text. | We have added data about the characteristics of families and people with mental health problems who participated in this study as well as diagnoses of people with mental disorders (Table 2). | Materials and methods  (Participants)  Row 193 - 194  And  Table 2  Row 200 – 201, Row 875 |
| Regarding Table 2 , it could be implemented with the percentages of the identified themes. | We have added the percentages of identified themes in Table 3 | Table 3  Row 877 - 878 |
| Row 184: The authors reported that they had administered the Self Reporting Questionnaire-29 (SRQ-29) scale to family members without any description; it would be useful to know which constructs/dimensions the questionnaire evaluates, related psychometric information and examples of items. In addition, it could be interesting to carry out regression analyses with the socio-personal data collected and questionnaire dimensions. | We identified the SRQ in the participants of this study using the SRQ-20. We apologize for the mistakes in writing our manuscript.  We performed an SRQ analysis and showed that all participants (25 families and 19 people with mental disorders) had scores below the cut-off point (<6).  We also performed correlation analysis using the Spearman Rank and showed that only the variable estimated time of home visit for cadres in a week had a significant relationship with SRQ (p-value 0.06). In contrast, age (p-value 0.081), gender (0.138), and length of time cadres interact with family and clients (0.278) did not significantly associate with the risk of mental-emotional problems.  We conducted regression analysis and obtained the model. After eliminating the length of time families interact with mental health cadres variables, the results revealed that the amount of Adjusted Coefficient of Determination (R2 adjusted) from this model, 13.3% of emotional mental health problems changes were related to the estimated time cadres make home visits in a week (p-value 0.044; B:-0.297) but not statistically significant with variable age (p-value 0.070; B:0.265). | Materials and methods  (Participants)  Row 226 - 242 |
| **‘Discussion and Conclusions section’** | | |
| In the discussion, as well as in the introduction, the authors should integrate the discussion and comment on the results by citing family psychoeducational approaches (1,2,3,4,5,6 listed below). | We have integrated the discussion section by citing literature following recommendations from reviewers. | Discussion  Row 533 – 536, Row 576 – 579, Row 590 - 597 |
| In the ‘Conclusions section’ the Authors should report how their study contributes to achieving the Global Goals of the 2030 Agenda for Sustainable Development. | We have inserted how our study contributed to SDGs 3:  This study contributes to achieving the Global Goals of the 2030 Agenda for Sustainable Development which was ensuring healthy lives and promoting well-being for all at all ages through the role of mental health cadres in supporting the care of patients with mental disorders and their families, thereby increasing family well-being and bridging mental health services for people with mental disorders. | Conclusions  Row 613 - 621 |
| **References** | | |
| References are reported inconsistently. For example, some contain the 'DOI' while others do not; some (i.e. citation 39) report volume and page numbers, others do not. Greater homogeneity and coherence between references would be needed | We have revised the inconsistently and made sure that references have homogeneity | References  Row 650 - 863 |
